# Comparison of calculated remnant lipoprotein cholesterol levels with levels directly measured by nuclear magnetic resonance

**DOI:** 10.1186/s12944-020-01311-w

**Published:** 2020-06-10

**Authors:** Jin Chen, Jie Kuang, Xiaoyu Tang, Ling Mao, Xin Guo, Qin Luo, Daoquan Peng, Bilian Yu

**Affiliations:** Department of Cardiovascular Medicine, the Second Xiangya Hospital, Research Institute of Blood Lipid and Atherosclerosis, Central South University, NO.139 Middle Renmin Road, Changsha, 410011 Hunan China

**Keywords:** Remnant cholesterol, Remnant lipoproteins, Nuclear magnetic resonance, Enzymatic method, Fasting state, Postprandial state, Residual risk, Atherosclerotic cardiovascular disease

## Abstract

**Background:**

Remnant cholesterol (RC) can partly explain the residual risk in atherosclerotic cardiovascular disease (ASCVD). A consensus method of measuring RC levels has not been established yet. In clinical practice, RC levels are usually calculated from the standard lipid profile, which are not true RC. Nuclear magnetic resonance (NMR) can measure RC levels directly. This study aimed to characterize RC at fasting and non-fasting states in more details and establish the performance of calculated RC and NMR-measured RC.

**Methods:**

Blood samples at fasting state and at 2 h and 4 h postprandial states were collected in 98 subjects. Lipid parameters including total cholesterol (TC), high-density lipoprotein cholesterol (HDL-C), low-density lipoprotein cholesterol (LDL-C), triglycerides (TG), subfractions 3, 4, and 5 of very low-density lipoprotein cholesterol (VLDL_3_-C, VLDL_4_-C, and VLDL_5_-C, respectively), and intermediate-density lipoprotein cholesterol (IDL-C) were measured by enzymatic method and NMR. RC levels calculated from the standard lipid profile or measured by NMR were referred here as RCe or RCn.

**Results:**

The RCe and RCn levels were different, but both of them increased after a meal (*P* < 0.05), especially at 4 h postprandial state. Low correlations were found between RCe and RCn in the 1st, 2nd, and 3rd quartiles of TG, but RCn showed great correlation with RCe in the highest quartile regardless of the fasting or non-fasting state (R = 0.611, 0.536, and 0.535 for 0 h, 2 h, and 4 h, respectively). However, across the 2nd and 3rd quartiles, RCe levels were nearly close to RCn levels. RCe levels tended to overestimate RCn levels in the 1st quartile of TGe levels with median differences of 0.23(− 0.13, 0.63) and underestimate RCn levels with median differences of − 0.23(− 0.33, 0.07) in the highest quartile of TGe levels.

**Conclusions:**

RC calculated from the standard lipid profile as TC minus LDL-C minus HDL-C is different from the NMR-measured RC. According to different TG levels, RC could overestimate or underestimate the actual RC level. Developing a consensus clinical method to measure RC levels is necessary, so that results from different studies and platforms can be more directly compared.

**Trial registration:**

Chinese Clinical Trial Registry, ChiCTR1900020873. Registered in 21 January 2019 - Retrospectively registered.

## Background

Atherosclerotic cardiovascular disease (ASCVD) is still a common cause of death worldwide [1, 2]. Up to now, lowering plasma levels of low-density lipoprotein (LDL) cholesterol (LDL-C) is one of the primary pharmacotherapies for primary and secondary preventions of ASCVD. However, substantial residual risk remains despite achieving optimal LDL-C levels and part of this residual risk could be explained by the cholesterol content of remnant lipoproteins (RLs), known as remnant cholesterol (RC) [3–5].

Chylomicrons (CMs) and very low-density lipoproteins (VLDLs) are triglyceride (TG)-rich and metabolized into smaller and relatively cholesterol-enriched particles by lipoprotein lipase once they are in the bloodstream [6]. These partially lipolyzed particles are known as RLs including chylomicron remnants (CM-Rs), VLDL remnants (VLDL-Rs), and intermediate-density lipoproteins (IDLs). RC was defined as the cholesterol content of RLs including denser subfractions of VLDL, IDLs in the fasting state, CM-Rs in the non-fasting state, as well as the altered fasting state in individuals with hypertriglyceridemia [7, 8]. Using direct measurements, RC was estimated to account for a large proportion of total cholesterol (TC) [9]. Elevated RC has a causal association with low-grade inflammation [10], which is related to unstable plaque and dysfunction of vascular endothelial cells. Moreover, studies have shown that high RC levels correlated with ischemic heart disease, high incidence of major adverse cardiovascular events, and high risk of ischemic stroke regardless of other risks [3, 4, 11, 12]. In addition to fasting RC level, non-fasting RC level has a consistent association with the risk for incident coronary artery disease (CAD).

Given their diversity in structure and composition, RLs are difficult to detect and isolate [13]. The traditional method for isolating subclasses of lipoproteins is ultracentrifugation [13]. In addition, high-performance liquid chromatography and immunoseparation assays using antibodies to apolipoprotein A and apolipoprotein B can separate RLs from the plasma directly [14, 15]. These methods are undoubtedly detailed in differentiating subclasses of lipoproteins and their lipid content. However, they are cumbersome and technically demanding, which limited their clinical application. Thus, a significant challenge in studying RC has been the absence of a consensus assay. Past studies have used differing methods for measuring RC, such as polyacrylamide gel electrophoresis, agarose gel electrophoresis, and vertical auto profile method, which uses ultracentrifugation with vertical rotor and single-density gradient spin [7, 8, 16–18]. In addition, Nordestgaard and Varbo [6, 19] pointed that the simplest way of defining RC is based on the cholesterol content of all TG-rich lipoproteins, that is, in the fasting state IDL and VLDL and in the non-fasting state CM-Rs. Using this definition, RC is the cholesterol content of all non-LDL and non-HDL and can easily be calculated from a standard lipid profile as total cholesterol (TC) minus LDL-C minus high-density lipoprotein cholesterol (HDL-C). This method can be used without extra cost in patients anywhere if standard lipid profile data are available. However, RC from this formula contains nascent VLDL cholesterol (VLDL-C) in addition to the true RC in the fasting state and includes CM cholesterol in the non-fasting state [20]. Therefore, it is necessary to find the relationship between the RC calculated from the standard lipid profile and the true RC.

The concept of using nuclear magnetic resonance (NMR) spectroscopy in lipoprotein measurement was first introduced in the early 1990s [21]. Over the past two decades, this technique has been widely used in cardiovascular research. NMR spectroscopy can be used to explore how cholesterol is distributed in a detailed continuum of lipoprotein fractions, which allows more accurate measurement of RC [9, 15, 22, 23]. In addition, the NMR method is an efficient and reproducible method and has high laboratory throughput [15].

To date, only one study has compared the agreement between RC acquired by clinical method and RC obtained by direct measurements, except for NMR [7]. Furthermore, most of the results were based on participants who underwent an oral fat tolerance test. Consequently, finding the relationship between RC obtained by clinical method and that measured by NMR based on participants with conditions close to real situations is important. In this study, RC was calculated from the lipid profile measured by standard laboratory procedures (RCe = TC − HDL-C − LDL-C) and measured by NMR (RCn = VLDL_3_-C + VLDL_4_-C + VLDL_5_-C + IDL-C) [7–9, 11, 24–26]. This study aimed to characterize fasting and non-fasting RC levels acquired by different clinical methods and to establish the performance of RCe vs RCn.

## Methods

### Study population

Using data from a trial registered at Chinese Clinical Trial Registry as ChiCTR1900020873, the current study assessed the association between calculated RC and NMR-measured RC. Details on objectives and methodological aspects were previously reported [27]. From June 2018 to December 2018, a total of 98 subjects at the Second Xiangya Hospital of Central South University were enrolled. Briefly, all these subjects underwent detailed clinical, laboratory, and angiographic examinations, and they were divided into the CAD group and the non-CAD group. The diagnosis of CAD was based on electrocardiographic changes, serum troponin T, and coronary angiography showing ≥50% stenosis in at least one main coronary artery. Non-CAD controls were recruited on the same period but free from atherosclerotic disorders as confirmed by coronary angiography or coronary computed tomography angiography. Besides, patients with significant hematologic disorders, infectious or inflammatory disease, various tumors, severe liver and/or renal insufficiency, severe uncontrolled diabetes or hypertension, and alcohol use or intensive exercise in the week before enrollment were excluded.

The study was approved by the Medical Ethics Committee of the Second Xiangya Hospital of Central South University (SXHCSU2019049). Informed consents were obtained from all subjects.

### Blood sample collection and lipid measurements

The blood sample collection method has been described previously [27]. Briefly, after overnight fasting for at least 10 h, all participants were allowed to have breakfast as usual. Chinese breakfast usually consists of steamed buns, noodles, eggs, milk, and soups with a bit of oil, and the energy content might be 500–600 kcal with 8–10% fat. The kinds of food and beverages consumed, the time of intake, quantities consumed in portion sizes, and preparation forms were recorded. Peripheral venous blood at the fasting state and at 2 h and 4 h postprandial states was collected. Blood samples were centrifuged immediately and analyzed or stored at − 80 °C for future analyses.

Blood lipids and lipoproteins were detected in two ways. First, the levels of plasma TC, HDL-C, LDL-C, and TG (TCe, HDL-Ce, LDL-Ce, and TGe, respectively) were measured by enzymatic measures using Roche automated clinical chemistry analyzer. Second, plasma TC, HDL-C, LDL-C, TG, VLDL3-C, VLDL4-C, VLDL5-C, and IDL-C levels (TCn, HDL-Cn, LDL-Cn, TGn, VLDL3-C, VLDL4-C, VLDL5-C, and IDL-C, respectively) were measured at ProteinT Biotechnology Co., Ltd. (Tianjin, China) using Bruker 600 M*Hz* Avance III NMR spectrometer as previously described [27, 28]. Details for the NMR experimental condition are provided in Additional File 1: Table S1. The spectra were normalized to the same quantitative scale using Bruker’s QuantRef manager within TopSpin which is based on the PULCON method; hence, the spectral intensity is normalized to proton concentration in units of millimoles per liter [29]. For data analysis, the study selected the commercial Bruker IVDr LIpoprotein Subclass Analysis (B.I.-LISA) method [28, 30] as lipoprotein distribution prediction method, which used a PLS-2 regression model as the algorithm for spectral deconvolution [31]. The PLS-2 model was built using bucketing parameters (size, number, and exclusions) similar way to those used by Okazaki et al. [31]. The lipoprotein subclass data available have five different VLDL subclasses: VLDL1 (average particle diameter of 64.0 nm), VLDL2 (53.6 nm), VLDL3 (44.5 nm), VLDL4 (36.8 nm), VLDL5 (31.3 nm), and IDL (28.6 nm) [32].

### Calculation of RC

The actual RC was defined as the sum of the cholesterol contents of the denser subfractions of VLDL and IDLs in the fasting state and CM-Rs in the non-fasting state. As described previously, VLDL can be identified into five subclasses by using NMR, namely, VLDL_1_ and VLDL_2_ as the large and buoyant TG-rich subclasses and VLDL_3–5_ as relatively cholesterol-rich small and dense subclasses [9, 24–26]. CM-Rs cannot be differentiated by NMR, and CM-Rs could be included in VLDL-Rs or even in IDLs, depending on their size [7, 9, 33]. Thus, the equation to calculate the actual RC by NMR (RCn) is as follows: VLDL_3_-C + VLDL_4_-C + VLDL_5_-C + IDL-C. In addition, in clinical practice, the estimated RC is calculated as follows: TC − HDL-C − directly measured LDL-C [7, 34]. Thus, in this study, another equation to calculate RC (RCe) is TCe − (HDL-Ce + LDL-Ce), which is calculated from the standard lipid profile measured by Roche automated clinical chemistry analyzers.

### Statistical analysis

All statistical data were analyzed by SPSS 25.0 (IBM Corp., Armonk, NY, USA) and Graph Pad Prism 7.0 software (GraphPad Software Inc., La Jolla, CA). Continuous variables approximating a normal distribution were reported as mean ± standard deviation (SD), and their differences were assessed by either Student’s t-test or analysis of variance methods. Continuous variables deviating from a normal distribution were reported as medians (25th–75th percentile) with differences compared by the Mann-Whitney U-test, Kruskal–Wallis test, or Wilcoxon signed rank test. Besides, categorical variables were expressed as count (%) and compared by χ^2^ test or Fisher’s exact test. The relationships among RC and other lipid parameters were assessed using Spearman’s correlations analysis.

In addition, the within-subject differences between RCe and RCn were calculated using the equation (RCe − RCn) / (RCn) [7]. Positive values represent overestimation of RCn by RCe, whereas negative values represent underestimation. Moreover, these differences within groups stratified by TG quartiles at different times and Spearman’s correlations between RCe and RCn in different TG quartiles were compared. An association between CAD and its risk factors was determined by logistic regression analysis. Two-tailed *P* values < 0.05 were considered statistically significant.

## Results

### Baseline characteristics

The baseline characteristics of the study population are illustrated in Table 1. The participants consist of 57 men (58%) and 41 women (42%), aged 38–66 years. As the chart shows, the CAD group was significantly older than the non-CAD group. The frequencies of other conventional risk factors such as male sex, hypertension, diabetes, and smoking habits were significantly higher in the CAD group than in the non-CAD group. In addition, baseline lipids were significantly different between the two groups; notable differences included much higher TGe, lower TCe and LDL-Ce, and higher HDL-Ce in the CAD group, and the lower LDL-Ce could be associated with the higher percentage of statin treatment in the CAD group. In terms of RC, the RCe level was slightly higher in the CAD group than in the non-CAD group [22.20(17.86, 28.67) mg/dL vs 18.15(14.19, 22.48) mg/dL, *P* = 0.003], and no significant differences were found in the RCn levels between the two groups.

**Table 1:** Baseline characteristics and lipoprotein summaries

Variable	All	Coronary artery disease		*P* value
	(*n* = 98)	Yes(*n* = 36)	No(*n* = 62)	
Age (years)	52 ± 14	61 ± 8	47 ± 14	<0.001 ^* b^
Men	57 (58%)	29 (81%)	28 (45%)	0.001^* a^
Hypertension	33 (34%)	18 (50%)	15 (24%)	0.009^* a^
Diabetes	14 (14%)	12 (33%)	2 (3%)	<0.001^* a^
Smoking	25 (26%)	14 (39%)	11 (18%)	0.021^* a^
BMI (kg/m2)	24 ± 4	25 ± 3	24 ± 4	0.605 ^b^
TC (mg/dL)	162.02 ± 35.13	147.32 ± 36.34	170.55 ± 31.67	0.001^* b^
LDL-C (mg/dL)	98.84 (77.99,120.46)	79.15 (69.98,111.58)	102.70 (89.38,122.30)	0.003^* c^
HDL-C (mg/dL)	41.62 ± 10.70	45.22 ± 10.23	35.42 ± 8.53	<0.001^* b^
TG (mg/dL)	130.97 (93.58,198.23)	166.37 (105.53,238.94)	120.35 (91.59,178.10)	0.047^* c^
RCe (mg/dL)	19.13 (15.35,24.32)	22.20 (17.86,28.67)	18.15 (14.19,22.48)	0.003^* c^
RCn (mg/dL)	21.51 (13.96,30.51)	21.09 (14.10,28.91)	21.51 (13.81,35.66)	0.517 ^c^

Values are mean ± SD, n%, or median (25th percentile, 75th percentile)

*BMI* Body mass index, *TG* Triglycerides, *TC* Total cholesterol, *HDL-C* High density lipoprotein cholesterol, *LDL-C* Low-density lipoprotein cholesterol, *RC* Remnant cholesterol

RCn = VLDL_3_-C + VLDL_4_-C + VLDL_5_-C + IDL-C; RCe = TCe minus (HDL-Ce + LDL-Ce)

^a^ χ^2^ test or Fisher’s exact test

^b^ Student’s T test

^c^ Mann-Whitney U-test

* *P*-value < 0.05

### NMR-measured baseline lipids showed significant positive correlations with those measured by enzymatic method

**Fig. 1:**
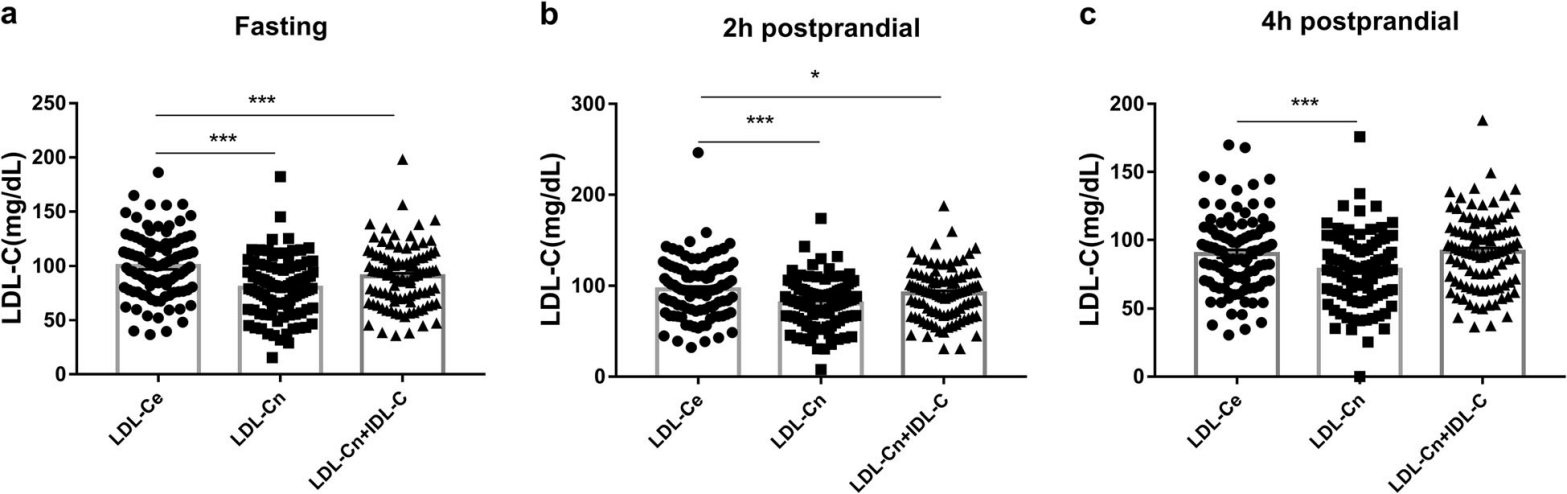
Comparisons between LDL-Ce levels and LDL-Cn levels or the sum levels of LDL-Cn and IDL-C. The sum levels of LDL-Cn and IDL-C = LDL-Cn + IDL-C; Fasting state(**a**), 2 h postprandial state (**b**), 4 h postprandial state (**c**). *** means *P* value < 0.001; ** means *P* value < 0.01; * means *P* value < 0.05

The correlations between lipid parameters measured by NMR and enzymatic method are shown in Table 2. By incorporating the data of all subjects into the statistical analysis, regardless of whether subjects are in the fasting or postprandial state, levels of plasma TGn were strongly correlated with TGe levels as expected (R = 0.979, 0.967, and 0.978 for 0 h, 2 h, and 4 h, respectively). Although the correlation value between TCn and TCe was > 0.9, LDL-Cn had a weaker correlation with LDL-Ce (R = 0.765, 0.777, and 0.744 for 0 h, 2 h, and 4 h, respectively). Notably, regardless of the fasting or postprandial state, the LDL-Cn levels were lower than the LDL-Ce levels (Fig. 1). To exclude the influence of IDL-C, it was necessary to compare LDL-Ce levels and the sum of IDL-C and LDL-Cn levels. Obviously, the sum of LDL-Cn and IDL-C levels were also lower than the LDL-Ce levels in fasting and 2 h postprandial states (*P <* 0.05).

**Table 2:** Spearman’s correlation coefficients between lipids measured by enzymatic method or NMR

	Spearman R (*P* value)	Slope	Intercept
**TGn vs TGe (*n*** **= 98)**			
Fasting	0.979(< 0.001 ^*^)	1.11 ± 0.02	−0.43 ± 2.90
2 h Postprandial	0.967(< 0.001 ^*^)	1.13 ± 0.02	2.05 ± 4.90
4 h Postprandial	0.978 (< 0.001 ^*^)	1.17 ± 0.03	−7.75 ± 5.83
**TCn vs TCe(*n*** **= 98)**			
Fasting	0.949 (< 0.001 ^*^)	0.94 ± 0.03	−0.06 ± 5.17
2 h Postprandial	0.934 (< 0.001 ^*^)	0.80 ± 0.03	17.31 ± 6.20
4 h Postprandial	0.937 (< 0.001 ^*^)	0.84 ± 0.03	10.61 ± 5.26
**HDL-Cn vs HDL-Ce (*n*** **= 98)**			
Fasting	0.858 (< 0.001 ^*^)	1.04 ± 0.05	−9.37 ± 2.56
2 h Postprandial	0.788 (< 0.001 ^*^)	0.91 ± 0.06	−4.71 ± 3.04
4 h Postprandial	0.700 (< 0.001 ^*^)	0.84 ± 0.07	1.16 ± 3.73
**LDL-Cn vs LDL-Ce (*n*** **= 98)**			
Fasting	0.765 (< 0.001 ^*^)	0.85 ± 0.07	31.93 ± 5.95
2 h Postprandial	0.777 (< 0.001 ^*^)	0.96 ± 0.07	18.83 ± 6.35
4 h Postprandial	0.744 (< 0.001 ^*^)	0.79 ± 0.07	27.79 ± 6.11
**RCn vs RCe (*n*** **= 98)**			
Fasting	0.586(< 0.001 ^*^)	0.45 ± 0.05	9.72 ± 1.39
2 h Postprandial	0.534 (< 0.001 ^*^)	0.46 ± 0.06	11.70 ± 1.66
4 h Postprandial	0.653 (< 0.001 ^*^)	0.98 ± 0.10	0.73 ± 2.97

Values are mean ± SD

*Indicates statistical significance *P* < 0.05

### Postprandial changes in blood lipids

**Fig. 2:**
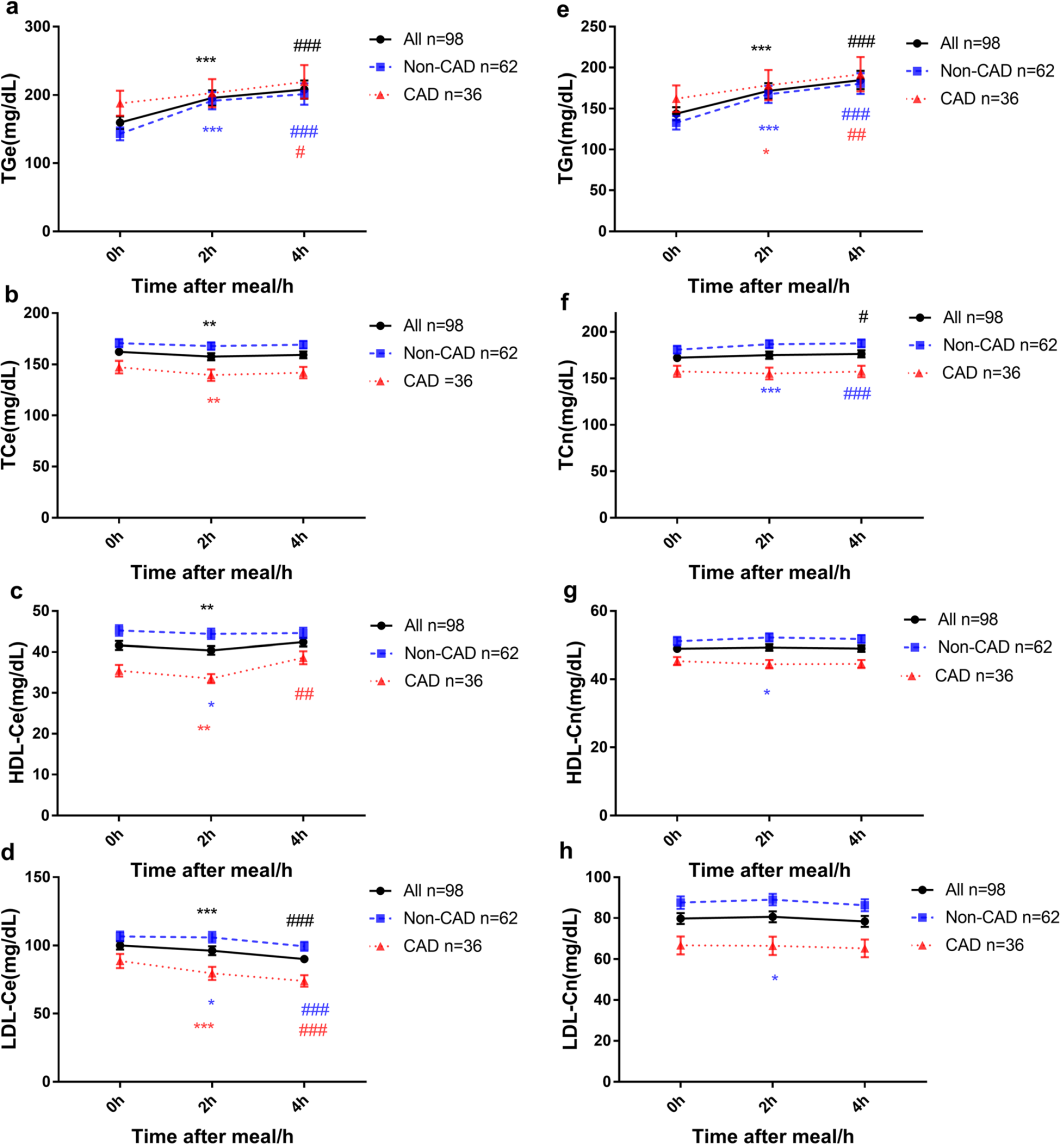
Changes in levels of blood lipids at 2 h and 4 h after a daily breakfast. Postprandial changes in levels of TGe (**a**), TCe (**b**), HDL-Ce (**c**), LDL-Ce (**d**), TGn (**e**), TCn (**f**), HDL-Cn (**g**), LDL-Cn (**h**) in different groups after a daily breakfast. Black indicate *P* value for all subjects; Blue indicate *P* value for non-CAD group; Red indicate *P* value for CAD group; When 2 h postprandial value compared with fasting value, *** means *P* value < 0.001, ** means *P* value < 0.01, * means *P* value < 0.05. When 4 h postprandial value compared with fasting value, ### means *P* value < 0.001, ## means *P* value < 0.01, # means *P* value < 0.05

In CAD or non-CAD participants, plasma TGe levels were significantly elevated after a meal as expected (Fig. 2a). Consistent with the previous reports [27], the levels of TCe, HDL-Ce, and LDL-Ce decreased significantly in the 2 h postprandial period. The 4 h postprandial TCe and HDL-Ce levels were nearly equal to their levels in the fasting state, while LDL-Ce levels were still decreased (Fig. 2 b-d). Similarly, the downward trends were seen in the TCn, HDL-Cn, and LDL-Cn levels in CAD patients after a meal. It appears that TCn, HDL-Cn, and LDL-Cn levels are increased in the 2 h postprandial state for non-CAD patients, but no statistical significance exists (Fig. 2 e-h).

**Fig. 3:**
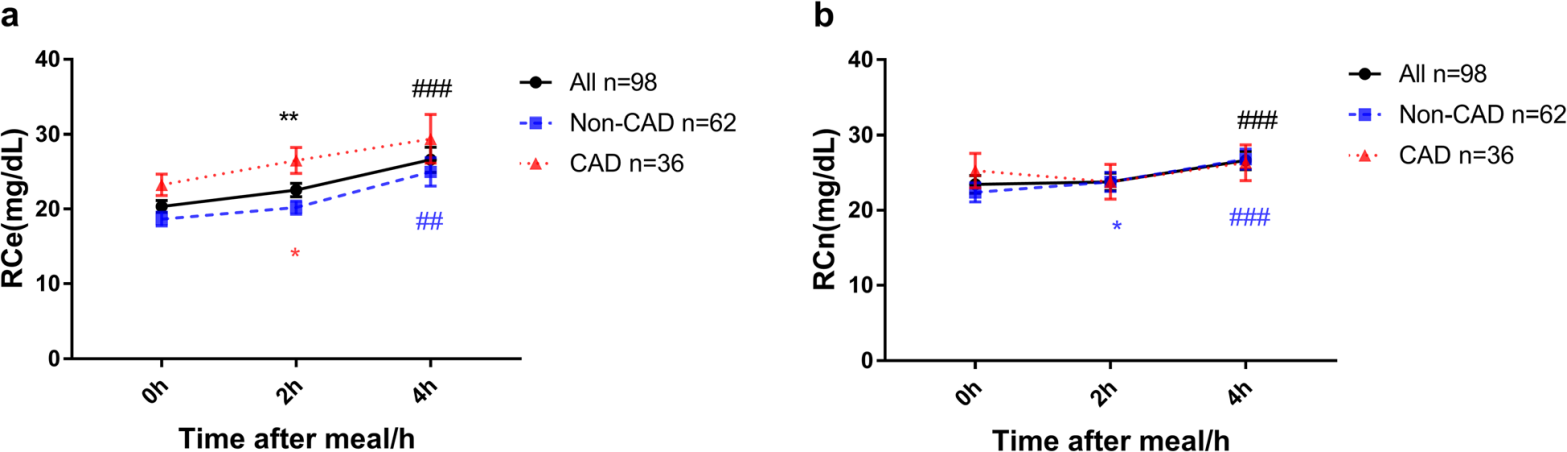
Changes in levels of RCe and RCn at 2 h and 4 h after a daily breakfast. Postprandial changes in levels of RCe (**a**), RCn (**b**), in different groups after a daily breakfast; Black indicate *P* value for all subjects; Blue indicate *P* value for non-CAD group; Red indicate *P* value for CAD group; When 2 h postprandial value compared with fasting value, *** means *P* value < 0.001, ** means *P* value < 0.01, * means *P* value < 0.05. When 4 h postprandial value compared with fasting value, ### means *P* value < 0.001, ## means *P* value < 0.01, # means *P* value < 0.05

Except for the slight decrease in RCn levels in CAD patients, regardless of dividing the subjects into the CAD group and non-CAD group, both RCe and RCn levels showed an upward trend after a typical breakfast (Fig. 3). Although the levels of TCe, HDL-Ce, and LDL-Ce decreased in the 2 h postprandial period, the total decrease in LDL-Ce and HDL-Ce levels was greater than that of the TC level (5.02 (− 1.16, 11.58) vs 4.05 (− 5.21, 9.65), *P* < 0.05), which could explain the increase in the calculated RCe level after a meal.

### Comparing different assessments of RC in the fasting and postprandial states

The correlations between RC and TG were first analyzed. As shown in Table 3, RCn and RCe both showed strong positive correlations with TG, and the correlation values between RCn and TGn were 0.847 for fasting and 0.765 and 0.827 for postprandial 2 h and 4 h, respectively, whereas the correlation values between RCe and TGe were 0.615 for fasting and 0.534 and 0.753 for postprandial 2 h and 4 h, respectively. RCn was strongly correlated with VLDL3-C, VLDL4-C, and IDL-C levels (fasting R = 0.899, 0.931, and 0.966; 2 h postprandial R = 0.853, 0.895, 0.969; 4 h postprandial R = 0.865, 0.883, and 0.962, respectively; Table 3). The concordance between RCe and VLDL3-C, VLDL4-C, or IDL-C was weaker (fasting R = 0.550, 0.501, and 0.579; 2 h postprandial R = 0.437, 0.425, and 0.531; 4 h postprandial R = 0.554, 0.502, and 0.658, respectively). However, VLDL5-C was weakly correlated with RCn (R = 0.435, 0.378, and 0.168 for 0 h, 2 h, and 4 h, respectively), even less so with RCe (R = 0.262, 0.221, and 0.057 for 0 h, 2 h, and 4 h, respectively). Moreover, although the calculation methods of the RCn and RCe were different, RCn still showed positive correlation with RCe at the fasting state (R = 0.586, *P* < 0.001, Table 2), and their correlation was slightly stronger after a meal (R = 0.653 for 4 h postprandial, *P* < 0.001).

**Table 3:** Spearman’s correlation coefficients between RC and other lipids parameters (*n* = 98)

	Fasting Spearman R (*P* value)		2 h postprandial Spearman R (*P* value)		4 h postprandial Spearman R (*P* value)	
	RCe	RCn	RCe	RCn	RCe	RCn
TGe	0.615 (< 0.001^*^)	0.848 (< 0.001^*^)	0.534 (< 0.001^*^)	0.759 (< 0.001^*^)	0.753 (< 0.001^*^)	0.801 (< 0.001^*^)
TGn	0.617 (< 0.001^*^)	0.847 (< 0.001^*^)	0.513 (< 0.001^*^)	0.765 (< 0.001^*^)	0.718 (< 0.001^*^)	0.827 (< 0.001^*^)
TCe	0.364 (< 0.001^*^)	0.536 (< 0.001^*^)	0.326 (< 0.001^*^)	0.652 (< 0.001^*^)	0.431 (< 0.001^*^)	0.651 (< 0.001^*^)
TCn	0.349 (< 0.001^*^)	0.582 (< 0.001^*^)	0.335 (0.001^*^)	0.690 (< 0.001^*^)	0.501 (< 0.001^*^)	0.717 (< 0.001^*^)
HDL-Ce	−0.270 (0.007^*^)	−0.296 (0.003^*^)	− 0.266 (0.008^*^)	− 0.136 (0.181)	− 0.267 (0.008^*^)	−0.241 (0.017^*^)
HDL-Cn	−0.141 (0.166)	−0.235 (0.019^*^)	0.024 (0.817)	0.045 (0.660)	0.014 (0.892)	−0.006 (0.952)
LDL-Ce	0.278 (0.006^*^)	0.588 (< 0.001^*^)	0.196 (0.055)	0.647 (< 0.001^*^)	0.117 (0.253)	0.504 (< 0.001^*^)
LDL-Cn	0.002 (0.986)	0.099 (0.330)	0.044 (0.672)	0.234 (0.020^*^)	0.073 (0.475)	0.189 (0.062)
VLDL3-C	0.550 (< 0.001^*^)	0.899 (< 0.001^*^)	0.437 (< 0.001^*^)	0.853 (< 0.001^*^)	0.544 (< 0.001^*^)	0.865 (< 0.001^*^)
VLDL4-C	0.501 (< 0.001^*^)	0.931 (< 0.001^*^)	0.425 (< 0.001^*^)	0.895 (< 0.001^*^)	0.502 (< 0.001^*^)	0.883 (< 0.001^*^)
VLDL5-C	0.262 (0.009^*^)	0.435 (< 0.001^*^)	0.221 (0.029^*^)	0.378 (< 0.001^*^)	0.057 (0.580)	0.168 (0.098)
IDL-C	0.579 (< 0.001^*^)	0.966 (< 0.001^*^)	0.531 (< 0.001^*^)	0.969 (< 0.001^*^)	0.658 (< 0.001^*^)	0.962 (< 0.001^*^)

*VLDL*_*3*_*-C* subfraction 3 of very low-density lipoprotein cholesterol, *VLDL*_*4*_*-C* subfraction 4 of very low-density lipoprotein cholesterol, *VLDL*_*5*_*-C* subfraction 5 of very low-density lipoprotein cholesterol, *IDL-C* Intermediate-density lipoprotein cholesterol

*Indicates statistical significance *P* < 0.05

To further investigate the relationship between RCe and RCn, this study analyzed the correlations between RCn and RCe stratified by TG quartiles at different time in all subjects. As shown in Table 4, low correlations were found between RCe and RCn in the 1st, 2nd, and 3rd quartiles of TGe, but RCn showed great correlations with RCe in the highest quartile regardless of the fasting or non-fasting state (R = 0.611, 0.536, and 0.535 for 0 h, 2 h, and 4 h, respectively, Table 4).

**Table 4:** The Spearman’s correlations between RCe and RCn according to the TGe quartiles

	Quartile 1	Quartile 2	Quartile 3	Quartile 4
**Fasting (*n*** **= 98)**	[54.87,93.58]	(93.58,130.97]	(130.97,198.23]	(198.23,426.55]
Spearman R (*P* value)	0.066 (0.760)	0.319 (0.120)	0.207 (0.320)	0.611 (0.002*)
Slope	−0.08 ± 0.24	0.27 ± 0.18	0.27 ± 0.16	0.56 ± 0.16
Intercept	17.06 ± 3.03	10.41 ± 3.68	15.49 ± 4.26	6.21 ± 6.05
**2 h Postprandial (*n*** **= 98)**	[46.02,110.40]	(110.40,173.01]	(173.01,258.19]	(258.19,606.19]
Spearman R (*P* value)	−0.025 (0.970)	−0.083 (0.669)	0.280 (0.175)	0.536 (0.007*)
Slope	−0.03 ± 0.19	0.11 ± 0.17	0.28 ± 0.20	0.63 ± 0.18
Intercept	17.22 ± 2.87	18.85 ± 3.49	16.00 ± 5.08	5.97 ± 6.98
**4 h Postprandial (*n*** **= 98)**	[44.25,110.40]	(110.40,168.14]	(168.14,269.69]	(269.69,694.69]
Spearman R (*P* value)	0.087 (0.693)	0.056 (0.790)	0.234 (0.260)	0.535 (0.007*)
Slope	0.09 ± 0.21	0.07 ± 0.22	0.19 ± 0.24	1.15 ± 0.42
Intercept	14.22 ± 3.45	17.80 ± 4.68	21.50 ± 6.57	−3.50 ± 18.40
**All statistics (*n*** **= 294)**	[44.25,105.31]	(105.31,157.08]	(157.08,232.74]	(232.74,694.69]
Spearman R (*P* value)	−0.021 (0.871)	0.106 (0.370)	0.200 (0.085)	0.549(< 0.001*)
Slope	0.01 ± 0.12	0.21 ± 0.12	0.19 ± 0.11	0.81 ± 0.17
Intercept	16.01 ± 1.68	14.59 ± 2.53	18.24 ± 2.88	2.98 ± 6.70

All statistics means incorporating all data of fasting, 2 h postprandial and 4 h postprandial RCe, RCn and TGe into statistical analysis

*Indicates statistical significance *P* < 0.05

Moreover, considering that 198.23 mg/dL might be a high cutoff value, which could bias the results, this study divided all subjects into two groups based on the normal value of the fasting TGe level: TGe-low group with < 150 mg/dL and TGe-high group with ≥150 mg/dL. Obviously, the ability for RCe levels to predict RCn levels was consistent with prior analysis, and significant correlations still exists between RCe and RCn in the TGe-high group; especially, their correlations were slightly stronger after a meal (R = 0.475, 0.527, and 0.567 for 0 h, 2 h, and 4 h, respectively; Table 5), but low correlations were found between RCe and RCn in the TGe-low group.

**Table 5:** The Spearman’s correlations between RCe and RCn according to TGe-low group and TGe-high group

	Fasting			2 h postprandial			4 h postprandial		
	Spearman R (*P* value)	Slope	Intercept	Spearman R (*P* value)	Slope	Intercept	Spearman R (*P* value)	Slope	Intercept
**All subjects (*n*** **= 98)**									
TGe-low group (< 150 mg/dL)	0.215 (0.111)	0.26 ± 0.10	12.31 ± 1.84	0.119 (0.475)	0.19 ± 0.17	15.47 ± 2.71	0.219 (0.181)	0.25 ± 0.17	12.47 ± 3.08
TGe-high group (≥ 150 mg/dL)	0.475 (0.001^*^)	0.43 ± 0.10	11.43 ± 3.33	0.526(< 0.001^*^)	0.49 ± 0.09	10.84 ± 2.97	0.567 (< 0.001^*^)	0.99 ± 0.17	0.94 ± 5.78
**Non-CAD (*n*** **= 62)**									
TGe-low group (< 150 mg/dL)	0.250 (0.115)	0.31 ± 0.12	11.10 ± 2.30	−0.081 (0.714)	−0.14 ± 0.24	19.44 ± 3.98	0.197 (0.357)	0.29 ± 0.21	10.77 ± 4.08
TGe-high group (≥ 150 mg/dL)	0.350 (0.210)	0.52 ± 0.18	6.30 ± 5.73	0.550(< 0.001^*^)	0.40 ± 0.11	10.73 ± 3.22	0.431 (0.008^*^)	0.74 ± 0.26	7.46 ± 8.79
**CAD (*n*** **= 36)**									
TGe-low group (< 150 mg/dL)	0.195 (0.487)	0.09 ± 0.21	15.41 ± 3.28	0.794(< 0.001^*^)	0.63 ± 0.14	11.69 ± 2.17	0.574 (0.025)	0.39 ± 0.30	11.89 ± 5.02
TGe-high group (≥ 150 mg/dL)	0.654 (0.001^*^)	0.36 ± 0.11	16.06 ± 3.78	0.673 (0.001^*^)	0.50 ± 0.14	15.48 ± 4.88	0.793(< 0.001^*^)	1.16 ± 0.20	−1.68 ± 7.46
**All statistics (*n*** **= 294)**	Spearman R (*P* value)			Slope			Intercept		
TGe-low group (< 150 mg/dL)	0.147 (0.091)			0.21 ± 0.08			13.75 ± 1.35		
TGe-high group (≥ 150 mg/dL)	0.526(< 0.001^*^)			0.68 ± 0.08			6.80 ± 2.67		

Values are mean ± SD

*CAD* coronary artery disease

All statistics means incorporating all data of fasting, 2 h postprandial and 4 h postprandial RCe, RCn and TGe into statistical analysis

*Indicates statistical significance *P* < 0.05

**Fig. 4:**
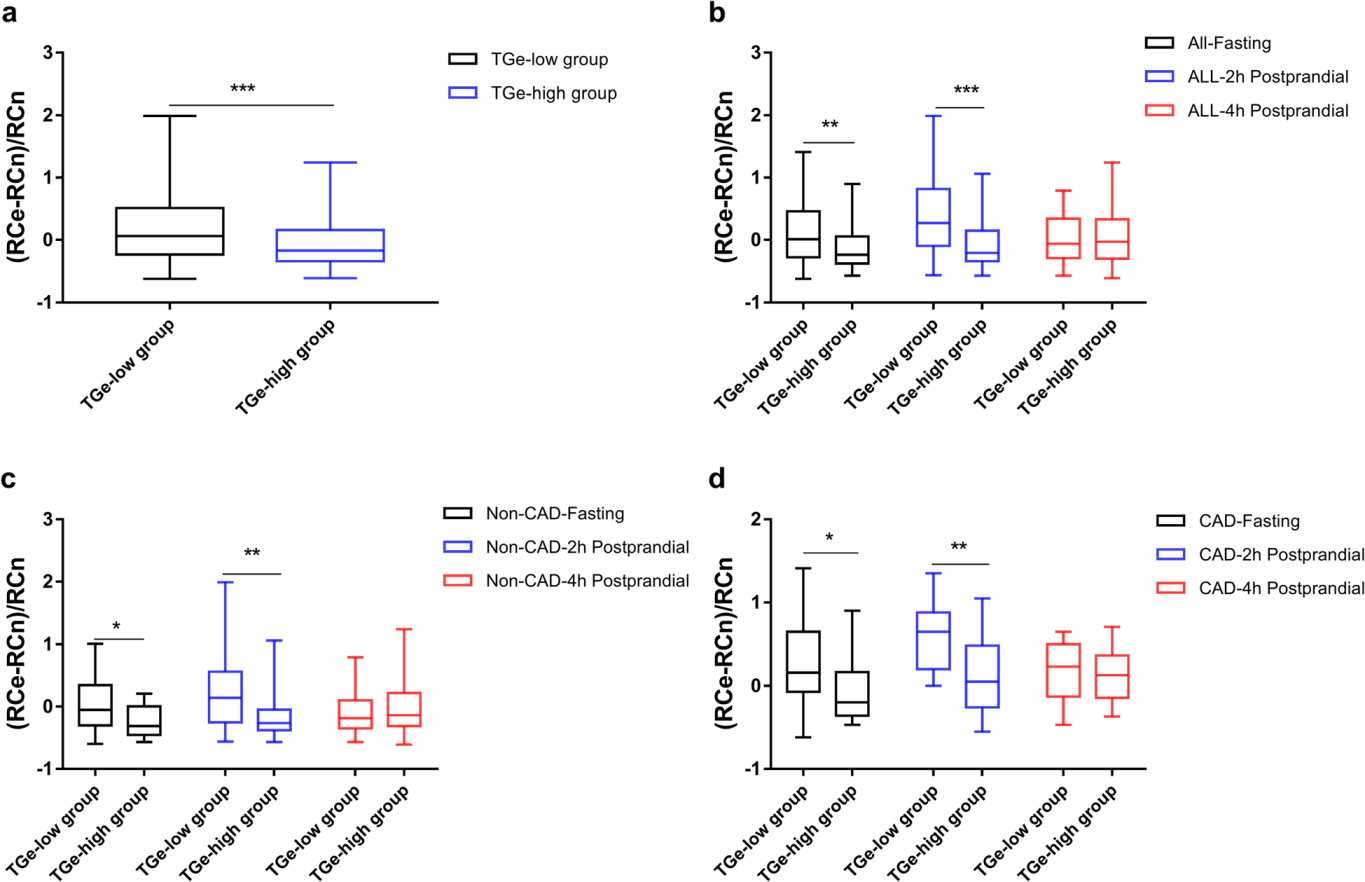
Comparisons between RCe and RCn in different groups at different time points. TGe-low group: < 150 mg/dL; TGe-high group: ≥ 150 mg/dL. (**a**) Incorporating all data of fasting, 2 h postprandial and 4 h postprandial RCe, RCn and TGe into statistical analysis; (**b**) in all subjects; (**c**) in non-CAD group; (**d**) in CAD group. *** means *P* value < 0.001; ** means *P* value < 0.01; * means *P* value < 0.05

**Fig. 5:**
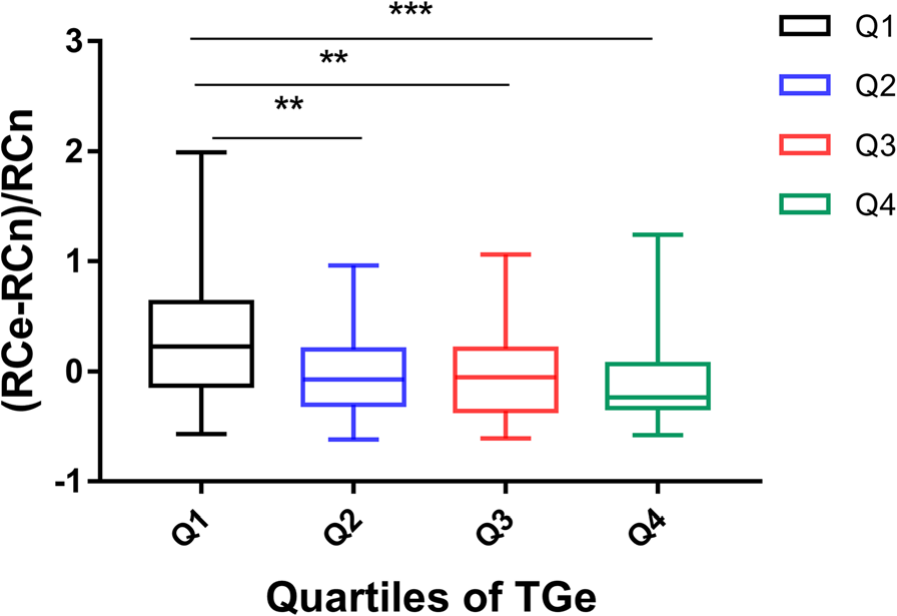
Comparisons between RCe and RCn at different TG levels in all subjects.(A) Incorporating all data of fasting, 2 h postprandial and 4 h postprandial RCe, RCn and TGe into statistical analysis, comparison between RCe and RCn in all quartiles of TGe, Q1: TGe ≤ 105.31 mg/dL, Q2: TGe = 105.32–157.08 mg/dL; Q3: TGe = 157.09–232.74 mg/dL; Q4: TGe > 232.74 mg/dL; *** means *P* value < 0.001; ** means *P* value < 0.01

To explore whether there is an exact TG cutoff value in which RCn levels are equal to RCe levels, the study compared the within-subject differences between RCe and RCn [(RCe-RCn)/RCn)] in the TGe-low and TGe-high group. Except for the 4 h postprandial state, regardless of the presence or absence of CAD, significant differences were found in (RCe-RCn)/RCn between the two groups (Fig. 4 and Additional File 1: Table S2). Moreover, this study incorporated all data of fasting, 2 h postprandial, and 4 h postprandial RCe, RCn, and TGe into the statistical analysis. As demonstrated in Fig. 5, the RCe levels were nearly close to the RCn levels across the 2nd and 3rd quartiles. Besides, the RCe levels tended to overestimate the RCn levels in the 1st quartile of the TGe levels with median differences of 0.23 (− 0.13, 0.63) and to underestimate RCn levels with median differences of − 0.23 (− 0.33, 0.07) in the highest quartile of the TGe levels.

Furthermore, logistic regression analysis was performed to assess the association between CAD and different assessments of RC. As shown in Additional File 1: Table S3, 2 h postprandial RCe (OR 1.54; 95% CI 1.12–2.12) and fasting RCn (OR 1.64; 95% CI 1.01–2.67) independently predicted CAD.

## Discussion

Up to now, this study is the first to compare RC measured by NMR (RCn) with RC calculated from the standard lipid profile using the eq. TC − LDL-C − HDL-C (RCe). Although notable discrepancies exist between RCe and RCn, the results showed that both RCe and RCn levels are significantly elevated after a meal in CAD and non-CAD participants. This study also highlights that RCe could overestimate or underestimate RCn according to different TG levels.

As shown in this study, RCe levels were not equal to RCn levels, but both of them increased after a meal. After a daily meal, CM and CM-Rs which are produced from the intestine appear in the bloodstream and reach the peak at 4 h postprandial [35, 36]. As a result of the increased liver synthesis and CMs competing for the same lipolytic pathway, levels of large VLDL also increase after a meal [37]. In this study, the RC levels at three time points including fasting, 2 h postprandial, and 4 h postprandial states were obtained. Similar to the TG levels, both RCe and RCn levels showed an upward trend lasting at least 4 h after a typical breakfast in CAD and non-CAD participants, except for the slight decrease in RCn levels in CAD patients. Although the phenomenon that RC levels increased after meals has been observed in previous studies [34, 38], the study participants were encouraged to choose their breakfast according to their dietary habits, so the results were closer to real situations. Although RC always has a great correlation with the risk of incident CAD, irrespective of the fasting or non-fasting state [3, 8, 11], the human body is in a non-fasting state most of the day. Therefore, compared with fasting RC levels, postprandial RC levels are more valuable for cardiovascular risk assessment.

Although using an estimate derived from a standard lipid profile to study RC is a convenient and intuitive approach, this method has limitations. This study used NMR-measured RCn and compared it with calculated RCe. Limited positive correlations between RCn and RCe were observed (R = 0.586, 0.534, and 0.653 for fasting, 2 h, and 4 h, respectively), which represent only half the variance with RCe. A previous study has also shown that RCe cannot reflect the true RC [7]. There are two main reasons for the discrepancies between RCe and RCn. First, Friedewald-estimated LDL-C or LDL-Ce includes IDL-C and is excluded from the RC calculated from the standard lipid profile [8, 9, 39]. Furthermore, the sum levels of LDL-Cn and IDL-C were smaller than the LDL-Ce levels in the fasting and 2 h postprandial states. This is because in addition to IDL-C, LDL-Ce also includes cholesterol in other lipoproteins, which is perhaps lipoprotein (a) [lp(a)] [8, 9]. Second, remnant size is important for atherogenicity. CMs and large VLDL are too large and cannot enter the arterial wall and probably do not cause atherosclerosis [40, 41]. However, small-to-medium-sized VLDL and CM-Rs are small enough to enter the arterial wall, but are too large to completely return to the bloodstream and thus are trapped inside the arterial wall [42–44]. Actually, RCe is the cholesterol cluster of all TG-rich lipoproteins, which include VLDLs, VLDL-Rs, IDLs, CMs, and CM-Rs. Unlike RCe, RCn is defined as the sum of the cholesterol contents of the densest VLDL-C subfraction and IDL-C measured by NMR, which is probably best used for smaller TG-rich lipoprotein particles that cause atherosclerosis. Unfortunately, so far, no studies compared the ability of RCe and RCn in predicting CAD risk. This study does not show that RCn has a stronger ability to predict CAD than RCe, which may be related to the small sample size. Further research with larger cohort is needed to address this issue.

Although calculated RC and NMR-measured RC are different, the results of this study suggest that the relationship between RCe and RCn is related to TG levels. Then, when their correlations stratified by TG quartiles were analyzed, it was found that the higher TG levels, the stronger correlation between RCe and RCn. Moreover, this study explored the correlations between RCe and RCn hierarchized by abnormal TG levels (150 mg/dL) at fasting state in clinic and found that RCe had low correlations with RCn when the TG levels were <  150 mg/dL. However, RCe showed positive correlations with RCn at high TG levels (≥150 mg/dL, R > 0.5). When the absolute values of RCe and RCn were compared, although a substantial discordance between RCe and RCn is further demonstrated, RCe levels are nearly equal to RCn levels within certain TG levels. These findings imply that using calculated RC instead of direct measurements to evaluate actual RC in research studies or clinical practice may be feasible. However, a larger sample study is needed to explore the relationship between their absolute values and find the cutoff value of TG.

### Study strengths and limitations

The present study has a number of strengths. This was the first study to explore differences and relationships between calculated RC and NMR-measured RC. The findings that RCe levels are nearly equal to RCn levels within certain TG levels proposed the clinical applicability of calculated RC. Besides, this study analyzed the results based on participants under daily life conditions which would provide more universal and generalizable results. Another strength of this study was the division of non-fasting state into 2 h and 4 h after a meal which was more detailed than previous studies. However, these findings should be interpreted in the context of several potential limitations. First, it is impossible to solely differentiate CM-Rs using the NMR spectroscopy method. Second, although a consensus method of measuring remnant lipoprotein levels is not established, numerous clinical studies have widely demonstrated that high RCe levels are a significant independent risk for ASCVD. Studies comparing RCs measured using other available methods are needed to determine additional discordance for further studies. Relevant studies are also needed to evaluate the association between different assessments of RC and ASCVD more accurately. In addition, the sample size is small and a further research in larger cohort is needed.

## Conclusions

In this study, both RCe and RCn levels were increased after a meal. These results imply that testing postprandial RC especially at 4 h to predict ASCVD risk is meaningful. Notable discrepancies exist between RCe and RCn, and RCe could overestimate or underestimate RCn according to different TG levels. Thus, it is necessary to develop a consensus clinical method for measuring RC levels, so that results from different studies and platforms can be compared more directly. This would be an important step in the study of RC, which might serve as a potential target for therapy in the future. Moreover, the phenomenon that RCe levels are nearly equal to RCn levels within certain TG levels imply that using calculated RC, which is a simple and convenient method of evaluating actual RC instead of direct measurements in research studies or clinical practice, may be feasible. However, a further research in larger sample is needed to find the TG cutoff value.

## Supplementary information

**Additional file 1: Table S1.** Experimental Parameters. **Table S2.** Values of (RCe-RCn)/RCn at fasting, 2 h and 4 h postprandial states according to TGe-low group and TGe-high group. **Table S3.** Logistic regression analysis for the association of RCe and RCn with CAD in fasting and non-fasting states (*n* = 98)

## Data Availability

The datasets used and/or analysed during the current study are available from the corresponding author on reasonable request.

## Abbreviations

ASCVD: Atherosclerotic cardiovascular disease
CAD: Coronary artery disease
CM-Rs: Chylomicron remnants
CMs: Chylomicrons
HDL-C: High-density lipoprotein cholesterol
IDL-C: Intermediate-density lipoprotein cholesterol
IDLs: Intermediate-density lipoproteins
LDL-C: Low-density lipoprotein cholesterol
NMR: Nuclear magnetic resonance
RC: Remnant cholesterol RC
RLs: Remnant lipoproteins
TC: Total cholesterol
TG: Triglyceride
VLDL_3_-C: Subfraction 3 of very low-density lipoprotein cholesterol
VLDL_4_-C: Subfraction 4 of very low-density lipoprotein cholesterol
VLDL_5_-C: Subfraction 5 of very low-density lipoprotein cholesterol
VLDL-Rs: VLDL remnants
