# Supplementary material for: Comparison of calculated remnant lipoprotein cholesterol levels with levels directly measured by nuclear magnetic resonance
Source: Lipids Health Dis. 2020 Jun 10;19:132. doi: 10.1186/s12944-020-01311-w (PMC7285517; doi:10.1186/s12944-020-01311-w)
Supplement: Supplementary file 1 — Additional file 1: Table S1. Experimental Parameters. Table S2. Values of (RCe-RCn)/RCn at fasting, 2 h and 4 h postprandial states according to TGe-low group and TGe-high group. Table S3. Logistic regression analysis for the association of RCe and RCn with CAD in fasting and non-fasting states (n = 98) [file 12944_2020_1311_MOESM1_ESM.docx]

**Table S1** Experimental Parameters

| **Parameters** |  |
| --- | --- |
| Pulse program | noesygppr1d |
| Time domain | 98304 |
| Dummy scans | 4 |
| Scans | 32 |
| Sweep width | 30 ppm |
| Acquisition time | 2.726 s |
| Relaxation delay | 4 s |
| Receiver gain | 90.5 |
| Dwell time | 27.7 µs |
| Mixing time | 0.01 s |
| Line broadening | 0.3 Hz |

**Table S2** Values of (RCe-RCn)/RCn at fasting, 2h and 4h postprandial states according to TGe-low group and TGe-high group.

|  | **(RCe-RCn)/RCn** | | |
| --- | --- | --- | --- |
|  | Fasting | 2h postprandial | 4h postprandial |
| **All statistics (294)** | | | |
| TGe-low group (< 150 mg/dL) | 0.06(-0.24, 0.51) | | |
| TGe-high group (≥ 150 mg/dL) | -0.17(-0.33, 0.16) | | |
| **All subjects (98)** |  |  |  |
| TGe-low group (< 150 mg/dL) | 0.01(-0.27, 0.46) | 0.76(0.42,1.05) | -0.05(-0.29, 0.34) |
| TGe-high group (≥ 150 mg/dL) | -0.24(-0.38, 0.05) | -0.21(-0.34, 0.15) | -0.30(-0.30, 0.33) |
| **Non-CAD (62)** |  |  |  |
| TGe-low group (< 150 mg/dL) | 0.74(0.63, 0.98) | 0.14(-0.25, 0.56) | -0.19(-0.35, -0.10) |
| TGe-high group (≥ 150 mg/dL) | -0.31(-0.46, 0.004) | -0.26(-0.38, -0.05) | -0.14(-0.31, 0.22) |
| **CAD (36)** |  |  |  |
| TGe-low group (< 150 mg/dL) | 0.16(-0.07, 0.65) | 0.65(0.20, 0.88) | 0.23(-0.13, 0.50) |
| TGe-high group (≥ 150 mg/dL) | 0.86(0.66, 1.10) | 0.05(-0.26, 0.49) | 0.12(-0.14, 0.37) |

Values are medians (25th–75th percentile). All statistics means incorporating all data of fasting, 2h postprandial and 4h postprandial RCe, RCn and TGe into statistical anlysis.

**Table S3** Logistic regression analysis for the association of RCe and RCn with CAD in fasting and non-fasting states (n=98)

|  | **OR** | **95% CI** | ***P* value** |
| --- | --- | --- | --- |
| Age | 1.13 | 1.02 – 1.26 | 0.022* |
| Gender (Male) | 330.72 | 3.76 – 29119.00 | 0.011* |
| Smoking | 3.11 | 0.17 - 58.79 | 0.449 |
| Hypertension | 5.12 | 0.37 – 71.81 | 0.225 |
| Diabetes | 64.38 | 1.27 – 3265.76 | 0.038* |
| Fasting RCe | 1.00 | 0.79 - 1.27 | 0.976 |
| 2h postprandial RCe | 1.54 | 1.12 – 2.12 | 0.008* |
| 4h postprandial RCe | 1.16 | 0.98 - 1.36 | 0.080 |
| Fasting RCn | 1.64 | 1.01 -2.67 | 0.045* |
| 2h postprandial RCn | 0.63 | 0.35 - 1.15 | 0.134 |
| 4h postprandial RCn | 1.05 | 0.61 - 1.79 | 0.868 |
| Fasting non-HDL-Ce | 1.17 | 0.94 – 1.45 | 0.168 |
| 2h postprandial non-HDL-Ce | 0.85 | 0.67 – 1.09 | 0.203 |
| 4h postprandial non-HDL-Ce | 0.92 | 0.79 – 1.08 | 0.318 |
| Fasting non-HDL-Cn | 1.06 | 0.80 – 1.41 | 0.681 |
| 2h postprandial non-HDL-Cn | 1.13 | 0.87 – 1.48 | 0.355 |
| 4h postprandial non-HDL-Cn | 0.81 | 0.61 – 1.08 | 0.144 |

OR: odd ratio; CI: conﬁdence interval. *Indicates statistical significance *P* < 0.05.
